# The saprotrophic dimension of *Exobasidium* (*Exobasidiales*, *Basidiomycota*): evidence for greater diversity and ecological flexibility than previously recognized

**DOI:** 10.3897/imafungus.17.180524

**Published:** 2026-03-16

**Authors:** Tereza Veselská, Tereza Ježková, Nombuso P. Ngubane, Adéla Wennrich, Martin Kostovčík, Denisa Hařovská, Martin Šigut, Petr Pyszko, Ryan Bracewell, Pavel Drozd, Dominik Begerow, Miroslav Kolařík

**Affiliations:** 1 Laboratory of Fungal Genetics and Metabolism, Institute of Microbiology of the Czech Academy of Sciences, Vídeňská 1083, 14000, Prague, Czech Republic Institute for Plant Science and Microbiology, Universität Hamburg Hamburg Germany; 2 Department of Biology, Indiana University, 1001 E 3rd St, Bloomington, IN 47405, USA Faculty of Science, University of Ostrava Ostrava Czech Republic; 3 Department of Genetics and Microbiology, Faculty of Science, Charles University, Viničná 5, 12000, Prague, Czech Republic Department of Biology, Indiana University Bloomington United States of America; 4 Institute for Plant Science and Microbiology, Universität Hamburg, Ohnhorststraße 18, 22609, Hamburg, Germany Faculty of Science, Charles University Prague Czech Republic; 5 Department of Biology and Ecology, Faculty of Science, University of Ostrava, 30. dubna 22, 70103, Ostrava, Czech Republic Institute of Microbiology of the Czech Academy of Sciences Prague Czech Republic

**Keywords:** Ecology, environmental communities, genome sequencing, GlobalFungi, plant pathogens, tree phyllosphere, *Exobasidium phylloplanum* sp. nov.

## Abstract

Fungi represent the largest group of plant pathogens, causing significant economic losses in agriculture and forestry worldwide. Species of the genus *Exobasidium* (*Exobasidiales*, *Basidiomycota*) are considered pathogens of plants from the order *Ericales*. While *Exobasidium* species notably impact tea and fruit production, their complete life cycles remain poorly understood, which hampers their management. These species are characterized by a dikaryotic parasitic stage tightly associated with living host tissues and a haploid, yeast-like saprotrophic stage. The prevalence, ecological significance, and potential contribution of this saprotrophic stage to the persistence of *Exobasidium* outside living host plants remain understudied. In this study, we confirmed the presence of several *Exobasidium* species in the leaf phyllosphere of five broad-leaved tree species in Central Europe using both cultivation and environmental DNA ITS2-based approaches. Additionally, we describe a new species without a known parasitic phase, *E. phylloplanum***sp. nov**., along with its physiological description and annotated genome. Environmental DNA surveys, using the GlobalFungi database, revealed that *E. phylloplanum* is not only common locally but also the most frequently detected *Exobasidium* lineage worldwide. This broad ecological amplitude contrasts with the narrow host specificity typical of parasitic congeners, suggesting that *E. phylloplanum* has adapted to a generalist saprotrophic life-history strategy. Our findings demonstrate that *Exobasidium* species can persist saprotrophically on diverse hosts, suggesting a broader ecological role and higher diversity than previously recognized. This research elucidates the diverse ecological roles of *Exobasidium* species and suggests that further genomic studies could reveal the genetic factors that underpin the different life strategies within this genus.

## Introduction

The genus *Exobasidium* Woronin (*Exobasidiales*, *Exobasidiomycetes*, *Basidiomycota*) comprises globally distributed biotrophic fungi that parasitize *Ericales* plants in three families, namely *Ericaceae* (Donk 1966; Begerow et al. 2002), *Symplocaceae* (Nagao et al. 2003, 2023), and *Theaceae* (Lee et al. 2015; Chaliha et al. 2021; Park et al. 2021). As a consequence of the biotrophic life-history strategy, *Exobasidium* species often show a high degree of host specificity (Nannfeldt 1981; Begerow et al. 2002; Brewer et al. 2014) and cause diverse symptoms, including leaf and fruit spots (e.g., *Exobasidium maculosum* on *Vaccinium*; Brewer et al. 2014), witches’ broom (e.g., *E. darwinii* on *Vaccinium reticulatum*; Piątek et al. 2012), or galls on leaves, stems, flowers, shoots, and buds (e.g., *E. japonicum* on *Azalea*; Graafland 1960).

Recently, *Exobasidium* species have attracted considerable attention due to the substantial economic losses they cause in tea and fruit production. For example, *Exobasidium vexans* reduces the quality of the tea crop, *Camellia sinensis* (Ponmurugan et al. 2016), resulting in a 40% decline in its global yield (Sen et al. 2020). Other species affect the productivity of blueberries and cranberries (Hildebrand et al. 2000; Ingram et al. 2019).

The genus comprises around 150 accepted species and varieties (Index Fungorum database, accessed 17 February 2026). Its life cycle is tightly linked to its hosts and has been well characterized in only a handful of species, such as *E. maculosum* (Ingram et al. 2019), *E. vaccinii* (Graafland 1960; Sundström 1964), and *E. vexans* (Sen et al. 2020). On a suitable host, basidiospores or conidia germinate either by budding or by producing a germ tube. In this phase, the fungus exists as haploid, saprophytic yeast-like cells or short hyphal segments. Subsequent conjugation between cells of compatible mating types results in a pathogenic dikaryotic mycelium. This mycelium penetrates plant tissues through an appressorium and grows intercellularly, forming a subepidermal hymenial layer. At this stage, characteristic disease symptoms become visible. Finally, the host epidermis ruptures, revealing the fungal hymenium, with basidia producing basidiospores and conidiophores producing conidia (Graafland 1960; Sundström 1964; Sen et al. 2020). Both types of propagules are then dispersed by wind (Sen et al. 2020) or insect vectors (Newell et al. 2023). Their overwintering phase and strategy are much less understood (Ajay et al. 2009; Ingram et al. 2019). This is especially puzzling given that, unlike their smut relatives, they lack a resting, thick-walled teliospore phase. From the available evidence, it appears that *E. vexans* overwinters within necrotic blisters in the form of mycelium and thick-walled, dormant basidiospores (Ajay et al. 2009), while *E. vaccinii* persists perennially within the host’s shoot and rhizome (Hildebrand et al. 2000). *Exobasidium maculosum* is thought to overwinter on the host plant surface saprophytically as yeast-like cells, which infect plant tissue in spring (Ingram et al. 2019).

Fungi from the subphylum *Ustilaginomycotina*, to which the genus *Exobasidium* belongs, are mostly known for their dimorphic life cycles (Begerow et al. 2000; Morrow and Frase 2009). During the saprotrophic phase, these fungi grow in a haploid yeast-like form not only on artificial culture media but also on their hosts and most likely also outside their hosts. In contrast, the pathogenic phase is characterized by the presence of filamentous dikaryotic mycelium growing within the host tissue (Begerow et al. 2014). Thus far, saprotrophic growth of *Exobasidium* outside their plant hosts has not been well documented. However, several studies have encountered *Exobasidium* spp. as part of phylloplane communities on diverse non-host plants (Inácio et al. 2005; Fonseca and Inácio 2006; Jumpponen and Jones 2010; Cordier et al. 2012; Nguyen et al. 2016; Cross et al. 2017; Qian et al. 2018; Ivashchenko et al. 2022; Marčiulynas et al. 2022; Menkis et al. 2022). While these findings are not definitive proof of active growth, they do suggest that *Exobasidium* may, during its yeast-like phase, be capable of living independently outside its host plant.

In the present study, we detail the saprotrophic life phase of *Exobasidium* species. During the study published by Šigut et al. (2022), we frequently detected *Exobasidium* in the phyllosphere communities of broad-leaved trees and in the guts of associated herbivorous insects. In the current study, we found that these *Exobasidium* sequences encompassed 12 distinct phylotypes, of which only seven clustered with described species with publicly available DNA barcodes. These observations suggest that the plant phyllosphere could host a hidden diversity of *Exobasidium* species with an unknown pathogenic phase during their saprotrophic growth. We further investigate the presence of a saprotrophic phase across all *Exobasidium* species with available DNA data by mining publicly available envDNA metabarcoding data in the GlobalFungi database (Větrovský et al. 2020) to identify occurrences beyond known host associations. We also describe a new *Exobasidium* species, *E. phylloplanum*, first encountered during metabarcoding of phyllospheric assemblages and subsequently isolated into pure culture from various healthy broad-leaved tree species and the guts of associated caterpillars. A pathogenic phase of this new *Exobasidium* species has not yet been identified. We propose that this species either has an extensive saprotrophic phase followed by a parasitic phase, causing mild or inconspicuous infection symptoms and thus escaping notice, or lives exclusively as a saprotrophic organism.

## Material and methods

### Assessment of *Exobasidium* diversity from environmental samples

In the spring of 2018, we sampled and analyzed microbiomes of the plant phyllosphere and the guts of associated herbivorous insects using DNA metabarcoding of the ITS2 region (Šigut et al. 2022). During that study, we revealed *Exobasidium* as a standard component of both microbiomes. In the present study, we explore and discuss this taxon in detail. Sampling and methods for DNA metabarcoding are described in Šigut et al. (2022). Briefly, we sampled leaves of five tree species (*Fagales*: *Alnus glutinosa*, *Carpinus betulus*, *Corylus avellana*, *Quercus petraea*, and *Q. robur*) and 20 species of leaf-chewer caterpillars (*Coleophoridae*: *Coleophora alnifoliae*, *C. flavipennella*; *Erebidae*: *Lymantria dispar*; *Geometridae*: *Agriopis aurantiaria*, *Operophtera brumata*, *Phigalia pilosaria*; *Gracillariidae*: *Phyllonorycter* sp., *Phy. coryli*, *Phy. esperella*, *Phy. nicellii*, *Phy. quercifoliella*, *Phy. roboris*, *Phy. tenerella*; *Noctuidae*: *Anorthoa munda*, *Orthosia cerasi*, *Or. cruda*; *Tenthredinidae*: *Fenusa dohrnii*; *Tischeriidae*: *Tischeria ekebladella*; *Tortricidae*: *Archips* sp., *Tortricodes alternella*). For detailed information, see Suppl. material 1. DNA was isolated from a whole leaf (containing both epiphytic and endophytic microbial communities) and a dissected caterpillar gut. In the current study, we included only samples from central Moravia (Střeň, 49°41.59'N, 17°08.39'E, 225 m a.s.l.), as only this locality had a comparable number of samples per tree. The selected locality is a riparian floodplain forest, a habitat where *Ericales* plants (the primary hosts of *Exobasidium*) are absent. Rarefaction analysis of amplicon sequence variant (ASV) tables was performed to assess dataset completeness and the appropriate data resampling level for comparative analysis. Specifically, the phyllosphere dataset was resampled to 2,500 reads and the caterpillar gut dataset to 400 reads (Suppl. material 2). The final phyllosphere dataset includes samples of *A. glutinosa* (72), *Ca. betulus* (72), *Co. avellana* (67), *Q. petraea* (71), and *Q. robur* (60). The gut dataset includes seven samples from larvae feeding on *A. glutinosa* and 12 samples from larvae feeding on *Ca. betulus*, eight samples from larvae feeding on *Co. avellana*, seven samples from larvae feeding on *Q. petraea*, and nine samples from larvae feeding on *Q. robur*.

Multivariate analyses were applied to infer relationships among *Exobasidium* spp. community structure and host plants. We first used detrended correspondence analysis (DCA) to estimate heterogeneity in species abundance along the gradient. After confirming the length of the gradient on the first DCA axis, principal component analysis (PCA) was performed on log-transformed data (log (*x* + 1)). To test a null hypothesis of no difference between the host plant species and *Exobasidium* spp. communities, an analysis of similarity (ANOSIM) was conducted on the dataset using the Jaccard and Morisita-Horn indices as measures of similarity, with 9,999 permutations. All statistical tests were performed in PAST v.5 (Hammer and Harper 2001).

### Isolation of *Exobasidium* species into pure cultures

*Exobasidium* species isolated in pure culture directly from caterpillar guts for further analysis. We were focused only on the species *Tischeria ekebladella*, with the known presence of *Exobasidium* in its microbiome (Šigut et al. 2022). *Tischeria ekebladella* was collected at the same site where metabarcode analyses were conducted (Střeň). In addition, we isolated *Exobasidium* from leaves of *Tilia cordata* (Slovakia, Marianka, 48°14.81'N, 17°04.03'E), another common tree species in temperate Europe. *Exobasidium* strains were isolated from the individually dissected caterpillar gut or from approximately 1 cm^2^ of *Tilia* leaf. Guts were smashed in 10 mL of sterile PBS buffer, and leaves were finely chopped with a sterile razor blade and transferred into 10 mL of sterile PBS buffer. One mL of the resulting mixture was transferred into a sterile Eppendorf tube and vortexed for 20 s. This mixture was serially diluted to 10^–1^ and 10^–2^ and plated on Petri dishes containing dichloran-rose bengal chloramphenicol agar (DRBC) supplemented with 0.2% malt extract (Oxoid), 2% urea, and 0.012% phenol red (Sigma-Aldrich, St. Louis, MO, USA). Urea and phenol red were added because *Exobasidium* exhibits urease activity (Sen and Komagata 1979) and turns the medium pink, helping distinguish it from other yeasts. All samples were incubated in two technical replicates at 25 °C for four weeks. The representative strains were deposited in the Culture Collection of Fungi (CCF) at the Department of Botany, Faculty of Science, Charles University, Prague, and in the Culture Collection of Yeasts (CCY) at the Institute of Chemistry, Slovak Academy of Sciences, Bratislava, Slovakia. Type material was deposited in the PRM herbarium in the National Museum, Prague.

### Molecular analyses of isolated strains

Genomic DNA was isolated from cultures growing on malt extract agar for 2–4 days at 25 °C using Chelex 100 Molecular Biology Grade Resin (Bio-Rad, Hercules, CA, USA) according to the protocol (Ferencova et al. 2017), with the modification that during the incubation, the samples were shaken on a Mixing Block (MB-102, BIOER) at 100 rpm. For molecular identification of strains, the internal transcribed spacer (ITS1, 5.8S, ITS2) and the large subunit of the rDNA (LSU, including the D1/D2 domain) regions were sequenced using primer pairs ITS1F (Gardes and Bruns 1993) and ITS4 (White et al. 1990) and LROR (Moncalvo et al. 1995) and NL4 (O’Donnell 1993), respectively (Piątek et al. 2012). PCR reaction volumes and cycling conditions followed those described by Kolařík et al. (2023). Purified amplicons were sequenced at Macrogen (Amsterdam, The Netherlands). The obtained sequences were aligned and manually edited in BioEdit 7.2.5 (Hall 1999) and deposited in NCBI GenBank under accession numbers (Table 1).

**Table 1.** List the best ITS BlastN matches of phylotypes from the envDNA metabarcoding of the tree leaf community.

Strain/ phylotype ID	BlastN best match. similarity (%), query cover (%)	Species identity
Phyl. 1	*Exobasidium* sp., MG813822, 99.5, 100.0, *E. gracile*, HQ398622, 99.5, 100.0	*E. gracile*/ *E. euryae*/ *E. camelliae*
Phyl. 2	Uncul., ON1225731, 100.0, 100.0, *E. bisporum*, AB180364, 99.0, 100.0	*E. bisporum*
Phyl. 3	Uncul., OP467200, 100.0, 100.0, *E. bisporum*, AB180364, 98.5, 100.0	*E. bisporum*
Phyl. 4	Uncul., MT241972, 99.5, 100.0, *E. bisporum*, AB180368, 98.4, 85.0	*E. bisporum*
Phyl. 5	*E. maculosum*, KR262420, 99.5, 100.0	*E. maculosum*
Phyl. 6	*Exobasidium* sp., OP374143, 100.0, 100.0, *E. rostrupii*, KR262425, 97.1, 88.0	*Exobasidium* sp.
Phyl. 7	*E. arescens*, FJ896135, 100.0, 100.0	*E. arescens*
Phyl. 8	Uncul., OP467309, 99.5, 100.0, *E. japonicum*, MW647952, 92.5, 100.0	*Exobasidium* sp.
Phyl. 9	Uncul., AM902052, 99.7, 100.0, *E. rhododendri*, CP110632, 96.2, 100.0	*E. phylloplanum*
Phyl. 10	Uncul., MG828026, 98.1, 100.0, *E. japonicum*, MW647952, 97.1, 100.0	*Exobasidium* sp.
Phyl. 11	*E. canadense*, EU692771, 93.6, 100.0, *E. otanianum*, AB180683, 93.2, 100.0, *E. nobeyamense*, AB180333, 93.6, 100.0	*Exobasidium* sp.
Phyl. 12	Uncul. KX195997, 100.0, 100.0, *E. miyabei*, AB180330, 99.5, 100.0	*E. miyabei*

### Phylogenetic analyses

Identification of reads obtained from DNA metabarcoding is described in Šigut et al. (2022). Seventy-five ASVs assigned to *Exobasidium* were filtered from the original dataset. This dataset was enriched with ITS sequences obtained from sequencing of *Exobasidium* isolated in pure culture in the present study, as well as with 50 sequences from 40 *Exobasidium* spp., obtained from GenBank. Sequences were selected to enable phylotype identification, so only those with ≥ 95% similarity to each phylotype were downloaded from NCBI GenBank (accessed 10 October 2023). ITS sequences of *Laurobasidium hachijoense* (*Cryptobasidiaceae*, *Exobasidiales*, *Exobasidiomycetes*) and *Tilletiopsis pallescens* (*Entylomatales*, *Exobasidiomycetes*) were included as outgroups. ASVs were clustered at a 99.5% similarity threshold with MAFFT v.7 (Katoh and Standley 2013), and a single ASV per cluster was retained as a representative. The reduced dataset comprised 78 sequences from the 26 *Exobasidium* ASVs identified in the present study, 50 representative ITS sequences, and the two outgroups (Table 2). The sequences were aligned using the online version of MAFFT v.7 with the default settings. The alignment was manually corrected in BioEdit v.7.2.5 (Hall 1999) (Suppl. material 3) and used as input for constructing a phylogenetic tree in IQ-TREE v.2.1.2 (Minh et al. 2020). The best DNA substitution models, GTR+G, TVEF, and K81UF+G for the ITS1, 5.8S rRNA gene, and ITS2 regions, respectively, were determined in PARTITIONFINDER v.2.1.0 (Lanfear et al. 2014) using the corrected Akaike information criterion. Phylogenetic trees were reconstructed in IQ-TREE using the maximum likelihood (ML) method and node support determined by nonparametric bootstrapping with 1,000 replicates. The graphical was output of the generated tree was produced in iTOL v.6 (Letunic and Bork 2021). The tree was rooted using *L. hachijoense* and *T. pallescens*.

**Table 2.** List of species used in molecular analyses with voucher/strain information, GenBank accession numbers, and references.

Species	Voucher/strain	ITS rDNA	LSU rDNA	Reference
*E. aequale*	LE F-332785	NA	PV961616	Dudka (2022)
*E. arescens*	TUB 015031	FJ896135	FJ896136	Piątek et al. (2012)
*E. bisporum*	IFO H-12021/IFO 9942	AB180364	AB177598	GenBank, submitted by H. Nagao in 2004
*E. bisporum*	IFO H-12038/ IFO 30152	AB180368	AB177596	GenBank, submitted by H. Nagao in 2004
*E. bisporum*	5426_356	OM614836	NA	Pavlov et al. (2023)
*E. camelliae*	EC (TUK)/MAFF 238578	AB180317	AB176712	GenBank, submitted by H. Nagao in 2004
*E. camelliae-oleiferae*	MAFF 239978	AB262798	AB262794	Nagao et al. (2009)
*E. canadense*	CGMCC 5.1647	EU692771	EU692791	Li and Guo (2010)
*E. caucasicum*	MAFF 238830	AB180682	AB178254	GenBank, submitted by H. Nagao in 2004
*E. cylindrosporum*	MAFF 238663	AB180357	AB177580	GenBank, submitted by H. Nagao in 2004
*E. cylindrosporum*	MAFF 238662	AB180356	AB177589	GenBank, submitted by H. Nagao in 2004
*E. darwinii*	TUB 019166	FJ896133	FJ896134	Piątek et al. (2012)
*E. dubium*	MAFF 238582	AB180319	AB177563	GenBank, submitted by H. Nagao in 2004
*E. empetri*	LE F-341345	NA	PV961619	Dudka (2022)
*E. euryae*	CGMCC 5.1316	EU692759	EU692779	Li and Guo (2010)
*E. expansum*	LE F-341346	PV961608	PV961620	Dudka (2022)
*E. ferrugineae*	BPI:882571	NR120076	JQ611711	Kennedy et al. (2012)
*E. formosanum*	CGMCC 5.1322	EU692775	EU692781	Li and Guo (2010)
*E. gracile*	AFTOL-ID 1643	DQ663700	DQ663699	Matheny et al. (2006)
*E. gracile*	515	KJ767650	KJ767651	Lee et al. (2015)
*E. gracile*	CB	HQ398622	NA	Peng et al. (2010)
*E. hemisphaericum*	AB177591	NA	AB177591	GenBank, submitted by H. Nagao in 2004
*E. rhododendri-siderophylli*	HMAS 183428	EU692765	EU692786	Li and Guo (2010)
*E. inconspicuum*	MAFF 238616	AB180347	AB177556	GenBank, submitted by H. Nagao in 2004
*E. japonicum*	MAFF 238176	AB180315	AB177548	GenBank, submitted by H. Nagao in 2004
*E. karstenii*	R.B. 2052	NA	AF487389	Begerow et al. (2002)
*E. kishianum*	MAFF 238624	AB180354	AB177555	GenBank, submitted by H. Nagao in 2004
*E. kunmingense*	CGMCC 5.1334	EU692763	EU692784	Li and Guo (2010)
*E. ledi*	52	ON117815	NA	Dudka (2022)
*E. lijiangense*	CGMCC 2.6921	NR_200499	OP470231	Jiang et al. (2024)
*E. lushanense*	CGMCC 5.1645	EU692767	EU692789	Li and Guo (2010)
*E. maculosum*	E12A1-1	NA	KF134405	Brewer et al. (2014)
*E. maculosum*	NCLC1-35	KR262384	NA	Stewart et al. (2015)
*E. maculosum*	D2-6	KR262420	NA	Stewart et al. (2015)
*E. maculosum*	E1-1	KR262421	NA	Stewart et al. (2015)
*E. maculosum*	C1-2	KR262409	NA	Stewart et al. (2015)
*E. miyabei*	MAFF 238595	AB180330	AB177579	GenBank, submitted by H. Nagao in 2004
*E. myrtilli*	R.B. 2055	NA	AF487390	Begerow et al. (2002)
*E. nobeyamense*	MAFF 238597	AB180331	AB177582	GenBank, submitted by H. Nagao in 2004
*E. noetherae*	BRIP 76280a	PQ061110	PQ047735	Tan and Shivas (2024)
*E. otanianum*	MAFF 238613	AB180345	AB177593	GenBank, submitted by H. Nagao in 2004
*E. oxycocci*	R.B. 2086	NA	AF487391	Begerow et al. (2002)
*E. pachysporum*	MAFF 238621	AB180352	AB177573	GenBank, submitted by H. Nagao in 2004
*E. pentasporium*	MAFF 238600	AB180334	AB177581	GenBank, submitted by H. Nagao in 2004
*E. perenne*	E81-3	NA	KF134418	Brewer et al. (2014)
*E. phylloplanum*	CCF 7021	PX591258	PX591251	This study
*E. phylloplanum*	CCF 6536	PV253747	PX591250	This study
*E. phylloplanum*	CCF 6537	PX591259	PX591252	This study
*E. pieridis*	MAFF 306193	NA	AB177575	GenBank, submitted by H. Nagao in 2004
*E. pieridis-ovalifoliae*	IFO9961	AB180367	AB177601	GenBank, submitted by H. Nagao in 2004
*E. pulchrum*	CGMCC 5.1652	EU692776	EU692795	Li and Guo (2010)
*E. reticulatum*	MAFF 239442	AB180377	AB180381	GenBank, submitted by H. Nagao in 2004
*E. rhododendri*	AFTOL-ID 1851	DQ667153	DQ667151	Matheny et al. (2006)
*E. rhododendri - russati*	HMAS 183433	EU692778	EU692797	Li and Guo (2010), This study
*E. rostrupii*	TUB 019165	FJ896132	FJ896137	Piątek et al. (2012)
*E. setsutaiense*	MAFF 247752	NR_199736.1	NG_244251.1	Nagao et al. (2025)
*E. shiraianum*	MAFF 238602	AB180336	AB177549	GenBank, submitted by H. Nagao in 2004
*E. siroboe*	MAFF 239964	LC656021	LC656026	Nagao et al. (2023)
*E. sundstroemii*	R.B. 2051	NA	AF487396	Begerow et al. (2002)
*E. symploci*	MES-1476	MK020095	NA	GenBank, submitted by M. E. Smith in 2018
*E. symploci-japonicae*	MAFF 238811	AB178255	NA	GenBank, submitted by H. Nagao in 2004
*E. uvae-ursi*	GLM-F105774	KY424482	NA	Kruse et al. (2017)
*E. vaccinii*	MAFF 238668	AB180362	AB177560	GenBank, submitted by H. Nagao in 2004
*E. vaccinii-uliginosi*	69	ON117816	NA	Dudka (2022)
*E. vexans*	TRISL_HY	MT581939	MT588787	GenBank, submitted by G. D. Sinniah in 2020
*E. woronichinii*	MAFF 238617	AB180348	AB177557	GenBank, submitted by H. Nagao in 2004
*E. yoshinagae*	MAFF 238606	AB180340	AB177551	GenBank, submitted by H. Nagao in 2004
*E.* sp. 1	2017.07.14_SC_05b	MW051432	NA	Jewell et al. (2021)
*E.* sp. 2	2017.06.30_AV_01	MW051431	NA	Jewell et al. (2021)
*E.* sp. 3	OTU1125 5.8S	MT594692	NA	Voyron et al. (2022)
*E.* sp. 4	C1-7 SH204830.06FU	LS421445	NA	GenBank, submitted by T. Jairuss in 2018
*Laurobasidium hachijoense*	MAFF 238665	AB180359	AB177562	GenBank, submitted by H. Nagao in 2004
*Tilletiopsis pallescens*	CBS 111622	AY259059	AY272004	Boekhout et al. (2006)

NA – not available.

To infer the phylogenetic position of *Exobasidium* strains isolated from leaves and insect material, we used both ML and Bayesian inference (BI) approaches. The dataset comprised concatenated ITS (ITS1, 5.8S, ITS2) and LSU rDNA sequences from the herein-isolated strains, representing all 56 *Exobasidium* species available in NCBI GenBank (accessed 19 February 2026). The sequences were treated as described above. The dataset had 62 sequences with 1,166 nucleotide sites, of which 620 were constant and 340 were parsimony-informative. ML analysis was conducted in IQ-TREE using the models GTR+G, TVMEF, K81UF, and GTR+I+G for ITS1, 5.8S, ITS2, and LSU, respectively. For Bayesian phylogenetic inference, a Markov chain Monte Carlo approach was applied in MRBAYES 3.2.7 (Ronquist et al. 2012) using the GTR substitution model with gamma-distributed rate variation and a proportion of invariable sites for all four partitions. The analysis ran for 2.5 million generations, with sampling every 1,000 generations, where the first 25% of samples were discarded as burn-in. The standard deviation of split frequencies was below 0.01.

### Biogeography assessment using published environmental sequences

The biogeography of *Exobasidium* species was assessed using the GlobalFungi v5.0 database (Větrovský et al. 2020), which contains fungal ITS1 and ITS2 sequences from envDNA metabarcoding studies, following the workflow described by Réblová et al. (2022). First, SEED v2.0.54 (Větrovský et al. 2018) was used to extract ITS1 and ITS2 regions from ITS sequences. Individual ITS1 and ITS2 sequences were then queried against the GlobalFungi database using the BLAST–group results search tool (accessed 18 December 2023). Metadata for 100% identical BLAST hits were used for further analysis. The worldwide distribution of the novel *Exobasidium* sp. CCF 7021 was plotted in *R* using the package ggplot2 v3.5.1 (Wickham 2016).

### Morphological, physiological, and biochemical characterization of *Exobasidium* sp. CCF 7021

*Exobasidium* sp. CCF 7021 (described in this paper as *E. phylloplanum* sp. nov.) strains were cultivated on malt extract agar (MEA, HiMedia, Mumbai, India) for seven days at 25 °C under ambient light. Morphological structures were observed in plate cultures as described by Cole et al. (1969). All three strains were physiologically characterized according to standard yeast taxonomic methods (Kurtzman et al. 2011). Optimal growth temperature was assessed by cultivation of strains at the following temperatures: 4, 6, 10, 20, 25, 30, 35, and 37 °C for 35 days on YM medium (malt extract 10 g/L (HiMedia, Mumbai, India), yeast extract 4 g/L, D-glucose 4 g/L, agar 10 g/L; pH 6.3). For the salinity tolerance analysis, the strains were grown at 25 °C for 35 days on YM medium supplemented with 0, 5, 10, and 16% (w/v) NaCl, respectively.

The ability to ferment or utilize different carbon sources was tested following the methods described in Wickerham and Burton (1948), Wickerham (1951), and Kurtzman et al. (2011). The ability of *Exobasidium* sp. CCF 7021 to ferment glucose and produce CO_2_ was tested against that of *Saccharomyces cerevisiae*, which was used as a positive control for fermentation. *Exobasidium vaccinii* (CBS 101459) was used as a positive control for glucose utilization, and a sterile medium was used as a negative control. Briefly, cultures were grown in triplicate in glass test tubes containing liquid yeast extract medium supplemented with glucose, with inverted Durham tube inserts. As an indicator for carbon utilization, bromothymol blue was used. The experiment was monitored every 12 h for the first week and then every 24 h for the following three weeks. Color changes in the medium and gas accumulation in the Durham insert were monitored.

Before the carbon utilization experiment, isolates were grown on a starvation medium of 10× Yeast Nitrogen Base (Sigma, Missouri, USA) without any carbon sources for 72 h. After this, the inocula were transferred onto 48-well plates containing 10× yeast nitrogen supplemented with different carbon sources. The tested carbon sources included D-glucose, D-galactose, D-mannitol, D-sorbitol, succinic acid, meso-erythritol, L-arabinose, D-xylose, D-maltose, citric acid, *p*-arbutin, D-salicin, D-cellobiose, starch, lactic acid, and D-ribose. Isolates of *Exobasidium* sp. CCF 7021 and *E. vaccinii* CBS 101459 (positive control) were tested in triplicate. For the sugars, yeast nitrogen base medium with 5% glucose was used as a positive control, since the ability of *Exobasidium* sp. CCF 7021 and *E. vaccinii* to utilize this sugar was demonstrated during the fermentation experiment. The plates were incubated at 22 °C. The density of yeast cells was checked once a week for eight weeks and scored following the guidelines of Kurtzman et al. (2011).

### Genome sequencing and annotation

The genome of *Exobasidium* sp. CCF 7021 was sequenced using the MinION sequencer (Oxford Nanopore Technologies, Oxford, UK). DNA was isolated from a haploid culture. High-molecular-weight DNA was extracted from isolate CCF 7021 using the Wizard® HMW DNA Extraction Kit (Promega, Wisconsin, USA) according to the manufacturer’s instructions, with minor adjustments. Specifically, all incubation steps were performed on the Thermomixer-Mixer HC (Starlab International GmbH, Hamburg, Germany) shaking at 300 rpm. Quantity and quality assessments were conducted on the resulting DNA product. The 260/230 and 260/280 ratios were measured using a BioPhotometer (Merck KGaA, Darmstadt, DE) to assess DNA purity. DNA quantity was assessed using the Qubit 4 Fluorometer and the associated 1× dsDNA assay kits (Thermo Fisher Scientific, Massachusetts, USA).

To prepare the genomic DNA library, the Native Barcoding Kit 24 V14 (SQK-NBD114.24, Oxford Nanopore Technologies, Oxford, United Kingdom) was used following the Native Barcoding Kit 24 v14 protocol (Oxford Nanopore Technologies, Oxford, UK). Based on the Qubit concentration values, 1,000 ng of DNA was transferred to a PCR tube for library preparation. The final library was prepared and loaded onto the MinION R10.4.1 flow cell on a MinION Mk 1B device for sequencing with settings selecting for a minimum read length of 1 kbp (Oxford Nanopore Technologies, Oxford, UK). Once sequenced, basecalling was performed using Dorado 7.4.13. Basecalling was conducted with chemistry set to DNA 400 bps–5 kHz and the basecalling configuration set to Super-High Accuracy.

The genome was *de novo* assembled using Flye 2.9.5 (Kolmogorov et al. 2019). The quality of assembly was assessed using QUAST 5.3.0 (Mikheenko et al. 2018) and BUSCO 5.8.0 (Manni et al. 2021) using the *Basidiomycota*_odb10 database. Repetitive sequences were identified by RepeatModeler 2.0.5 (Flynn et al. 2020) and then masked by RepeatMasker 4.1.5 (https://repeatmasker.org/). The masked genome was annotated with BRAKER2 (Brůna et al. 2021) using a concatenated dataset comprising proteins from 18 publicly available annotated *Exobasidiomycetes* genomes sourced from NCBI (for details, see Suppl. material 5). Carbohydrate-active enzymes (CAZymes) were annotated using dbCAN3 (Zheng et al. 2023) with HMMER, Diamond, and dbCAN-sub for CAZyme family annotations. Predictions obtained from at least two tools were retained. Signal proteins were first predicted with SignalP 6.0 (Teufel et al. 2022). Signal proteins with transmembrane helices were detected with Phobius 1.01 (Käll et al. 2004) and DeepTMHMM 1.0 (Hallgren et al. 2022), and proteins with GPI anchors were identified using PredGPI (Pierleoni et al. 2008). These proteins were excluded from further analyses. Localization of remaining proteins was inferred using DeepLoc 2.1 (Ødum et al. 2024); only proteins with predicted extracellular localization were further used as input for analysis of putative effectors using EffectorP 3.0 (Sperschneider and Dodds 2022). A BlastP search against the PHI-base database v.4.18 (Urban et al. 2025) was used to track genes involved in pathogen–host interactions. Chromosome synteny among *Exobasidium* species was drawn in the *R* package Genespace 1.4 (Lovell et al. 2022) using OrthoFinder 2.5.4 (Emms and Kelly 2015) and MCScanX (Wang et al. 2012). Additional whole-genome-sequenced *Exobasidium* species, *E. cylindrosporum* YG638 (Bioproject no. PRJNA833290, Liu et al. 2023), *E. maculosum* A7-4 (PRJNA442817, JGI Genome Portal, unpublished), *E. rhododendri* CBS101457 (PRJNA863915, Li et al. 2023), and *E. vaccinii* MPITM (PRJNA196015, JGI Genome Portal, unpublished), together with the most closely related species from *Exobasidiales* (Wang et al. 2015), *Acaromyces ingoldii* MCA 4198 (JGI Genome Portal, unpublished) and *Meira miltonrushii* MCA 3882 (JGI Genome Portal, unpublished), were included in the comparative genomic analysis. The genome assemblies of these species were retrieved from public databases, NCBI and JGI, respectively, and processed following the same analytical workflow as applied to *Exobasidium* sp. CCF 7021, beginning with repeat masking.

## Results

### Diversity of *Exobasidium* in plant phyllosphere and insect gut from phylogenetic analysis of envDNA data

**Figure 1.**
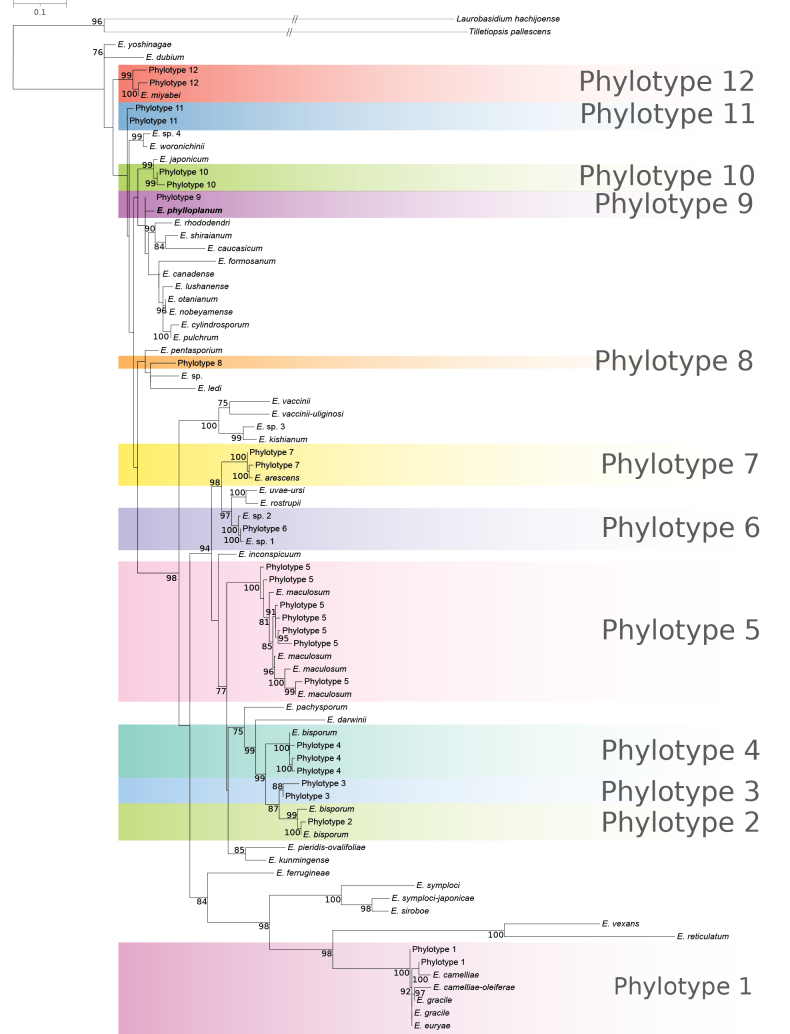
A maximum likelihood phylogenetic tree inferred from the ITS region. The phylogenetic tree was built from the ITS1, 5.8S, and ITS2 rDNA sequences of *Exobasidium* species available in the NCBI database, plus the ITS2 metabarcode sequences from our study. Bootstrap support higher than 75% is indicated for the branches. The hatch marks on the branches indicate that they were shortened to 1/3 of their original length for presentation purposes.

We detected 39 distinct ASVs from leaf material, totaling 4,036 reads, and 36 ASVs from insect material, totaling 880 reads, corresponding to 0.47% and 5.10% of the total number of fungal reads in the respective datasets (Suppl. material 2). Phylogenetic analysis of the ITS region clustered these ASVs into 12 phylotypes (Fig. 1, Table 1). Seven of these phylotypes grouped with already described species such as *E. arescens*, *E. bisporum* (covering three revealed phylotypes), *E. camelliae*/*E. camelliae-oleiferae*/*E. euryae*/*E. gracile*, *E. maculosum*, and *E. miyabei*. The best-hit search generated by the NCBI BLASTN tool (Altschul et al. 1997) revealed that the other five phylotypes have similarity below 94% to the described species in the NCBI database (Table 1). Thus, these phylotypes belong to so far unsequenced or undescribed taxa.

### Distribution and frequencies of *Exobasidium* in host trees

Alpha diversity of the *Exobasidium* communities was assessed using the Shannon (H’) and Simpson indices (D). The lowest alpha diversity was recorded in *Q. robur* (mean H’ = 0.32; mean D = 0.17). This was followed, in order of increasing diversity, by *A. glutinosa*, *Co. avellana*, *Ca. betulus*, and *Q. petraea*. The highest alpha diversity was observed in *Quercus petraea* leaves (mean H’ = 0.50; mean D = 0.39) (Suppl. material 6).

**Figure 2.**
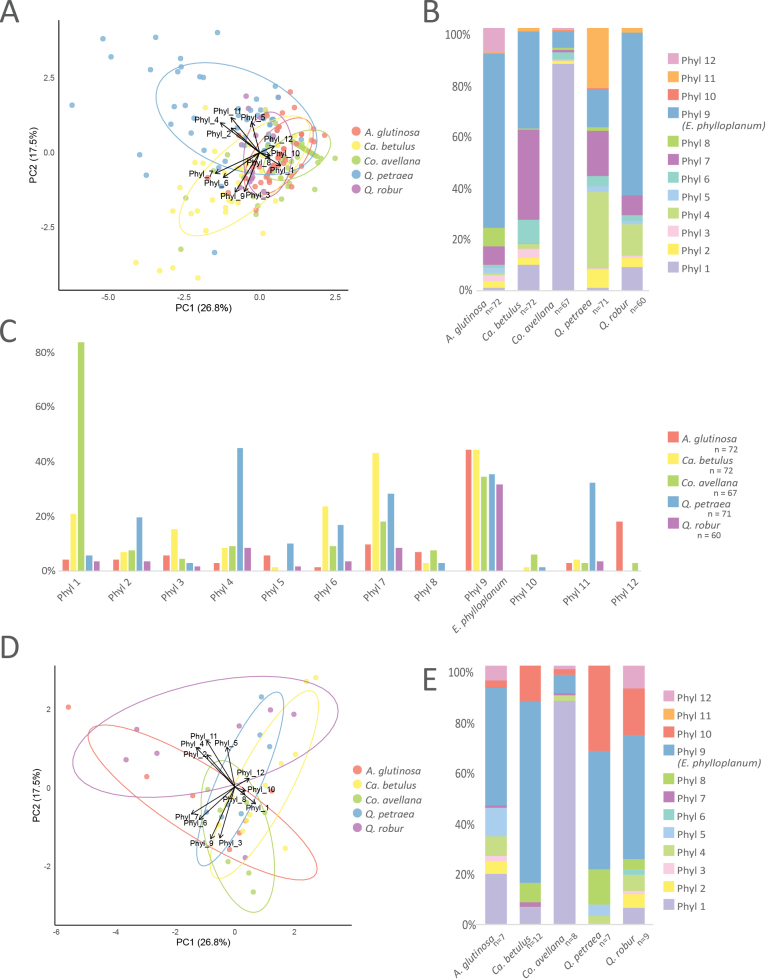
Distribution of 12 *Exobasidium* phylotypes across the five analyzed tree species. **A**PCA analyses of *Exobasidium* community in tree phyllosphere. **B** Relative abundance of phylotypes in the phyllosphere, based on sequencing read counts. **C** Occurrence frequency of phylotypes per host tree species, expressed as the percentage of leaf samples in which each phylotype was detected. *Exobasidium phylloplanum* (Phyl 9) was the most widespread phylotype, occurring in > 30% of samples from all hosts. In contrast, Phyl 1 showed a strong host preference, detected in > 80% of *Corylus avellana* samples. **D**PCA analyses of *Exobasidium* community in caterpillar guts. **E** Relative abundance of phylotypes in the guts of leaf-feeding caterpillars, based on sequencing read counts.

The ANOSIM analysis significantly separated individual tree *Exobasidium* phyllosphere communities (*p* < 0.02). The only exception was the community of *A. glutinosa*, which did not differ significantly from that of *Q. robur* (*p* = 1) (Suppl. material 7). The PCA analysis clearly separated the community of *Q. petraea*, characterized by the presence of phylotypes 2, 4, 5, and 11, and that of *Ca. betulus*, distinguished by phylotypes 3, 6, 7, and 9. The community of *Co. avellana* was defined by phylotypes 1 and 10 (Fig. 2A). The proportions of individual *Exobasidium* phylotypes in the phyllosphere communities of analyzed tree species are shown in Fig. 2B. The community of *A. glutinosa* was dominated by phylotype 9 (described in this study as *E. phylloplanum*), which represented more than 66% of *Exobasidium* reads. The community of *Co. avellana* was dominated by phylotype 1, accounting for nearly 87% of *Exobasidium* reads. *Quercus petraea* hosted diverse *Exobasidium* phylotypes, with phylotypes 4, 7, 9, and 11 being the most common, while phylotype 12 was absent. *Quercus robur* was dominated by phylotype 9, which made up 62% of all *Exobasidium* reads. Phylotypes 9 and 7 were the most common on *Ca. betulus*, where they represented 71% of all *Exobasidium* reads. Some *Exobasidium* phylotypes were enriched in a particular tree species over the others, whereas others occurred equally in the analyzed tree phyllospheres (Fig. 2C). The most striking examples of tree preference were phylotypes 1, 4, and 11, occurring in 90% of *Co. avellana* samples, 45% of *Q. petraea* samples, and 32% of *Q. petraea* samples, respectively.

### Distribution and frequencies of *Exobasidium* in insect guts

The ANOSIM and PCA analyses did not detect any significant differences between datasets (Fig. 2D, Suppl. material 7). All phylotypes detected on leaves were also found in insect guts, except phylotype 11, which was not detected (Fig. 2E). Their proportions also largely reflected those found in the phyllosphere of the respective trees. However, phylotypes 7 and 4 were less abundant in insect gut communities, possibly because they were unable to survive the harsh gut environment. Surprisingly, phylotype 10, which was rare in phyllosphere communities, was abundant in the intestinal communities of insects feeding on *Q. petraea*, accounting for 33% of *Exobasidium* reads. Phylotype 9 had the highest proportion in both the phyllosphere and gut *Exobasidium* communities, except in *Co. avellana*, where phylotype 1 was the most abundant in both habitats.

### Biogeography assessment using published environmental sequences

**Figure 3.**
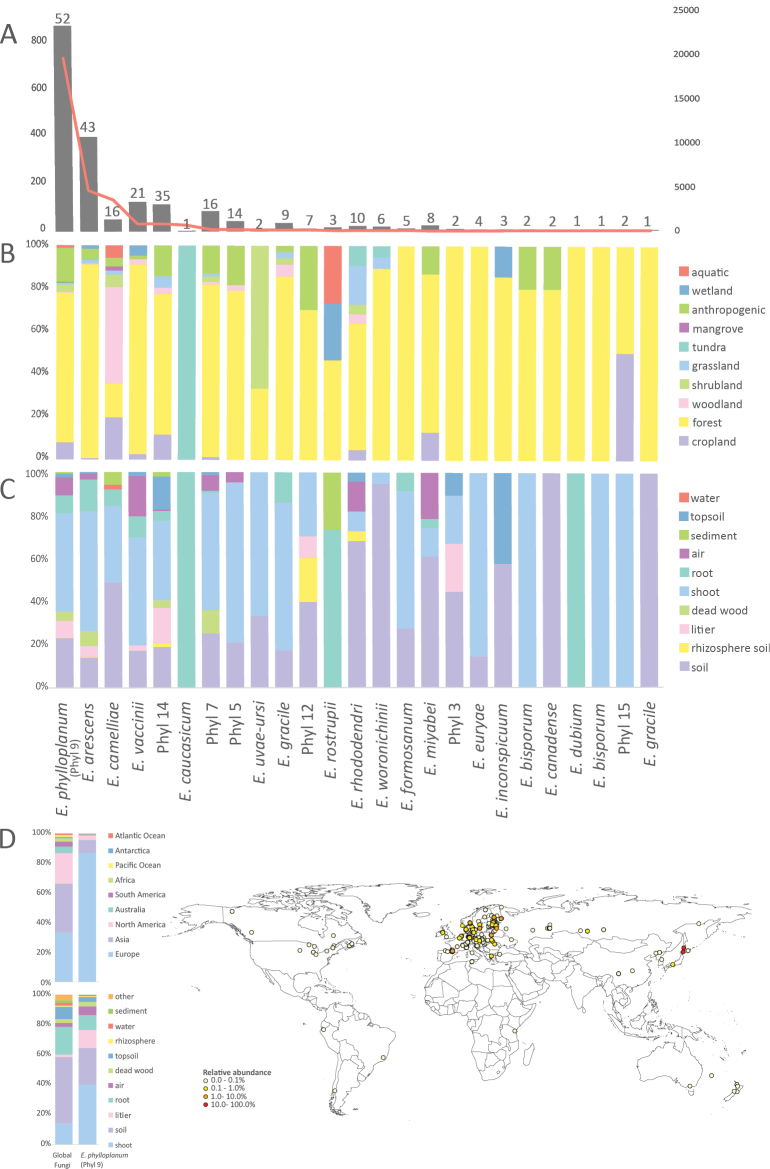
Distribution of *Exobasidium* lineages (i.e., species or phylotypes) in envDNA samples included in the GlobalFungi database. **A** Absolute abundance. Grey bars indicate the number of samples containing each lineage (left axis); the red line shows the corresponding number of sequencing reads (right axis). Numbers above the bars denote the number of independent studies reporting each lineage. *Exobasidium phylloplanum* (i.e., Phyl 9) is the most common taxon, followed by *E. arescens*, *E. camelliae*, and *E. vaccinii*. **B** Distribution of *Exobasidium* lineages across biomes based on relative sample counts. **C** Distribution of *Exobasidium* lineages across substrates based on relative sample counts. *Exobasidium* is primarily associated with soils, shoots, and roots sampled in the forest biome. **D** Geographical distribution and substrate affinity of *E. phylloplanum* versus all taxa present in the GlobalFungi database. The geographical distribution of individual *Exobasidium phylloplanum* samples is shown on the map, along with relative abundance (i.e., the proportion of reads) in each sample.

From the 40 analyzed *Exobasidium* species/phylotypes, 17 were encountered in at least one environmental sample (Suppl. material 8). Of these, *E. phylloplanum* (i.e., phylotype 9) was the most frequently detected phylotype (Fig. 3A). This species was found in 841 samples from 52 different studies. It mainly occurred in data from European forests, particularly plant shoots and soil. Notably, the center of distribution is in temperate regions, with an absence in the otherwise well-surveyed boreal and Mediterranean areas (Fig. 3D). Surprisingly, the well-known plant parasites *E. arescens*, *E. vaccinii*, and *E. camelliae* were also common in environmental samples (Fig. 3). Both *E. arescens* and *E. vaccinii* were found on plant shoots and roots in European forests, as well as in soil. *Exobasidium vaccinii* was also found in air samples. The primary environmental distribution for *E. camelliae* is in Asia, followed by North America. This species was mainly associated with plant shoots and woodland soils. Another noteworthy discovery was the distribution of phylotype 3 (belonging to the *E. bisporum* clade) and phylotype 12 (belonging to the *E. miyabei* clade). Exact hits for both phylotypes were higher in the environmental samples than in the reference Sanger sequences of *E. bisporum* and *E. miyabei*. Phylotype 12 was present in 111 samples (from 35 studies), whereas *E. miyabei* was present in only 23 samples (from eight studies). Phylotype 3 was found in 83 samples from 16 studies, whereas *E. bisporum* (AB180364/AB177598) was only present in five samples from two studies. *Exobasidium bisporum* (AB180368/AB177596) was even rarer, encountered only in two samples, both from a single study. Phylotype 5, which clustered with *E. maculosum*, was present in 43 samples from 14 studies, whereas the *E. maculosum* NCBI reference sequence had no hits in the GlobalFungi database. *Exobasidium* phylotypes identified in the present study showed low similarity to known species. Phylotypes 6, 11, and 8 were rarely detected in environmental samples, most often in forest ecosystems, particularly in root and soil samples (Fig. 3B, C). We did not find any exact hits for phylotype 10 in the environmental samples.

### Isolation of *Exobasidium* from leaf and insect material into pure culture

*Exobasidium* sp. CCF 7021, identical to the ITS sequence of phylotype 9 and described below as *E. phylloplanum*, was the only *Exobasidium* species isolated from healthy plant leaves and caterpillar guts. Two strains of *E. phylloplanum* were isolated from the guts of the caterpillars *Tischeria ekebladella*, and one strain was isolated from *Tilia cordata*.

### Phylogenetic analysis

**Figure 4.**
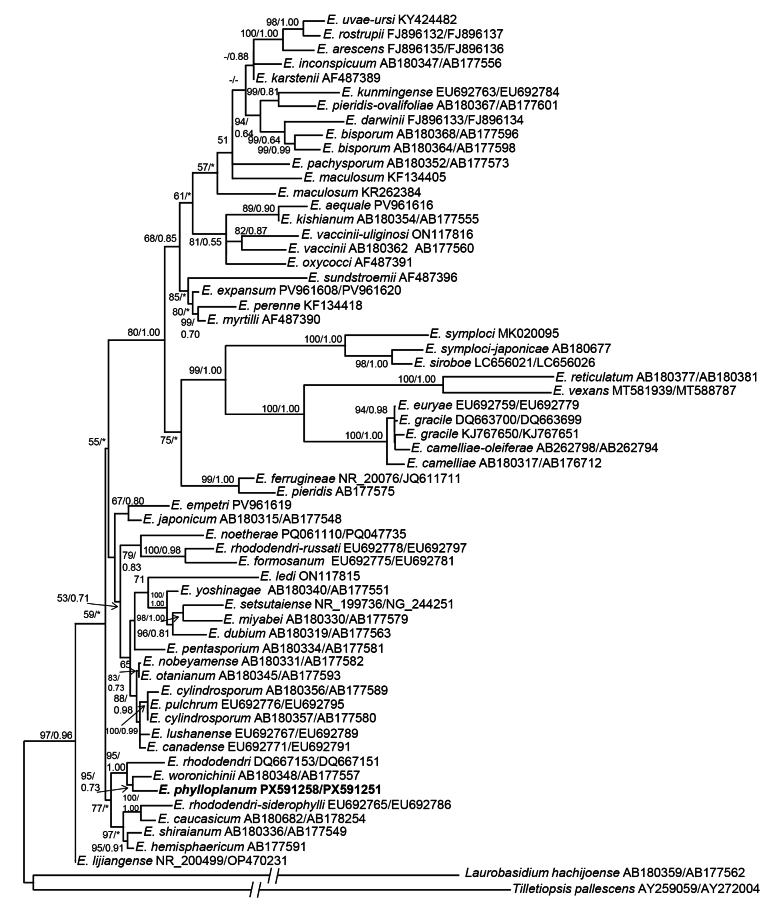
A phylogeny of the genus *Exobasidium* based on ITS and LSU rDNA sequences. **A** Maximum likelihood phylogenetic tree is presented. The numbers next to the internal nodes are maximum-likelihood bootstrap and Bayesian MCMC posterior probabilities; values ≥ 50/0.5 are shown. The GenBank accession numbers for ITS and LSU rDNA sequences are shown after the taxon name. The branch leading to the outgroups, *Tilletiopsis* and *Laurobasidium*, was shortened to 1/3 of their original length for presentation purposes.

Phylogenetic analyses placed *Exobasidium* sp. CCF 7021 as a sister species to *E. woronichinii* and *E. rhododendri* (Fig. 4). *Exobasidium* sp. CCF 7021 differs from *E. woronichinii* (AB180361, LC672666) by 3.9% and 1.8% nucleotides in the ITS and LSU regions, respectively. All three strains of *Exobasidium* sp. CCF 7021 were identical.

### Genome analysis

The genome of *E. phylloplanum*CCF 7021 was assembled into five nuclear scaffolds with a total genome size of 17.7 Mb, with N50 and L50 values of 5.8 Mb and 2, respectively, and one mitochondrial scaffold with a length of 34,792 bp. The average coverage depth was 122, with 93% of reads mapped back to the genome. BUSCO completeness was 95.5%. Repetitive elements constituted 1.56% of the genome. We identified 7,463 proteins. The results are summarized in Table 3.

**Table 3.** Detailed statistics and BUSCO results for genome assembly and annotation of *Exobasidium phylloplanum*.

Assembly statistics and annotation	
Genome size (Mb)	17.7
Number of scaffolds	5
GC (%)	44.93
N50 (Mb)	5.8
L50	2
Mapped (%)	93
Avg. coverage depth	122
BUSCO (%)	95.4
TE (%)	1.56
Number of annotated proteins	7,463

We found high chromosome synteny among *E. cylindrosporum*, *E. rhododendri*, and *E. phylloplanum* (Fig. 6A), in which contigs 3 and 4 are collinear with chromosome 3 in the congeners. Comparative analysis with other parasitic *Exobasidium* species and related taxa, *Acaromyces ingoldii* and *Meira miltonrushii*, revealed that all species have a similar number of genes coding for CAZymes (Fig. 6B, F), ranging from 224 in *E. vaccinii* to 268 in *E. maculosum*, most of which belong to the GH and GT classes. A similar pattern was found in genes involved in pathogen–host interactions, where the least number of genes was found in *E. rhododendri* (2,808) and the most in *E. maculosum* (3,699) (Fig. 6C, F). The genome of *E. maculosum* was enriched in signal proteins, including putative effector genes (Fig. 6D), while the genome of *E. phylloplanum* was enriched in genes coding for secondary metabolites, mainly belonging to polyketide synthases (T1PKS) (Fig. 6E). Detailed information about gene counts is available in Suppl. material 11.

### Taxonomy of *Exobasidium* sp. CCF 7021

*Exobasidium* sp. CCF 7021 (i.e., phylotype 9) was identified by molecular and culture methods as a standard component of the microbial communities of tree phyllospheres, especially those of *A. glutinosa* and *Q. robur*, and of the gut of the caterpillar *Tischeria ekebladella*. Additionally, an isolate of this species was obtained from leaves of healthy *Tilia cordata* in Slovakia. A biotrophic phase of *Exobasidium* sp. CCF 7021 has not yet been observed. Although we cannot exclude the possibility that this lineage corresponds to a historically described but unsequenced species, several independent lines of evidence support its recognition as a distinct taxon, including its consistently high environmental frequency in Central Europe and its phylogenetic position within the *Rhododendron*-specific lineage (see Discussion for further explanation).

#### Exobasidium phylloplanum

Taxon classificationAnimaliaExobasidialesExobasidiaceae

M. Kolařík, Ježková, Ngubane, Veselská sp. nov.

9E30D2DD-02BB-5D04-AFC8-071F541BA483

861482

Fig. 5

##### Etymology.

The name *phylloplanum* consists of the words *phyllos* (Greek) = leaf and *planum* (Latin) = plain, reflective of its association with the phylloplane.

##### Diagnosis.

Colonies on MEA with distinct bright pigments (orange, brown, red-brown, pink). Conidia fusiform, 8.2 ± 3.0 µm in length and 1.8 ± 0.2 µm in width. Sexual state not known. Genetically (rDNA), it differs from the closest species, *E. woronichinii*, by 3.9% and 1.8% nucleotides in the ITS and LSU regions, respectively (Fig. 4).

##### Typus.

SLOVAKIA • Bratislava region, Marianka, 48.246833°N, 17.067167°E, alt. 228 m; isolated from phylloplane of *Tilia cordata*; 6. Nov. 2022; T. Ježková (***holotype*** PRM 964352, dried culture CCF 7021, ***isotype*** PRM 964353, culture ex-type CCF 7021, CCY 102-1-1). GenBank sequences: ITS = PX591258, LSU = PX591251, SSU = PX591256; Whole genome sequence: – PRJEB104467 UNITE database: SH0934703.10FU.

##### Description.

**Figure 5.**
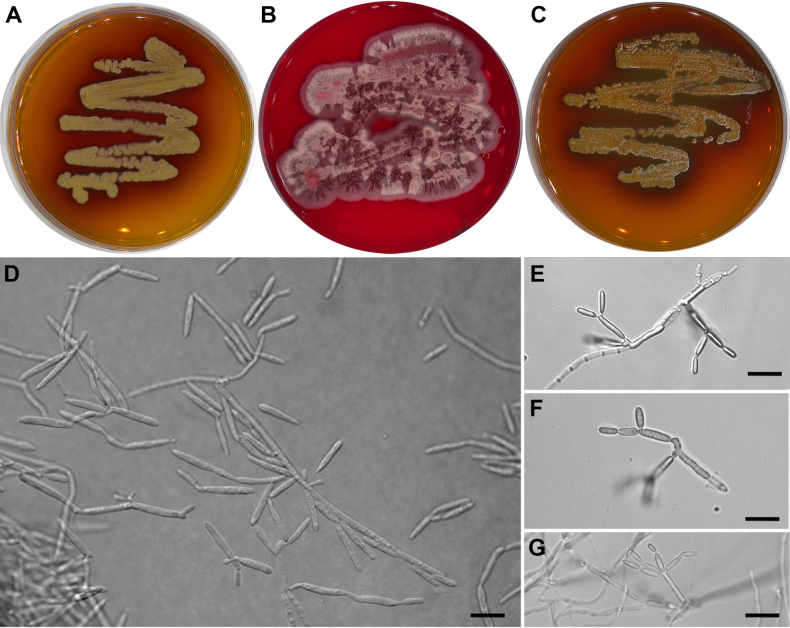
Morphology of *Exobasidium phylloplanum*. Colonies on MEA after three weeks at 25 °C, **A**CCF 6536. **B**CCF 6537. **C**CCF 7021. **D–G** Micromorphology of CCF 7021 showing fusiform blastoconidia formed acropetally in branched or unbranched chains on sterigmata-like structures, bars = 10 µm (**D**, **E**, **F**, **G**).

Cultures on MEA (3 d old) exhibited filamentous growth with formation of fusiform blastoconidia that were formed acropetally in branched or unbranched chains on sterigma-like structures. Mean **conidia** length was 8.2 ± 3.0 µm and width 1.8 ± 0.2 µm (Fig. 5). After one week, colonies achieved a diameter of 0.4 ± 0.04 cm; after two weeks, 0.8 ± 0.3 cm; and after three weeks, 0.9 ± 0.2 cm (Suppl. material 9). The pigmentation of colonies became visible after one week of cultivation. In the second week, the pigmentation diffused into the growth medium. The pigmentation of mature colonies is orange-brown in strains CCF 6536 and CCF 7021, while CCF 6537 shows pink-violet pigmentation.

**Figure 6.**
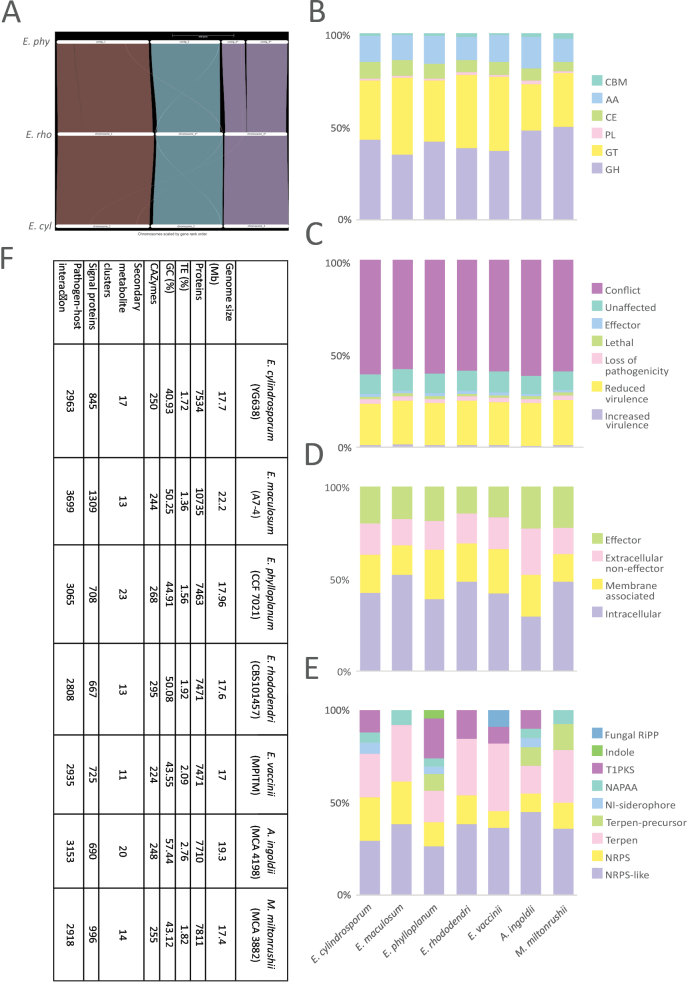
Comparative genomic analysis. **A** High chromosome synteny was revealed among three *Exobasidium* species with chromosome or near-chromosome assembly available. Contig 3 and 4 of species *E. phylloplanum* appear collinear with chromosome 3 in congeners. Contig 5 was not included in the analysis as it has no annotated protein. **B** Annotation of CAZymes classes. **C** Genes involved in pathogen–host interactions. **D** Signal proteins. **E** Annotation of secondary metabolite clusters revealed expansion of T1PKS gene clusters in *E. phylloplanum*. **F** Genomic features of *Exobasidium* species and *Acaromyces ingoldii* and *Meira miltonrushii*.

##### Physiological and biochemical tests.

Grows at temperatures between 6–25 °C, with the optimal growth temperature between 20–25 °C. Increasing salinity in the growth medium decreases the growth potential of *E. phylloplanum*. Salinity levels of 16% or higher entirely impede *E. phylloplanum* growth. Based on the fermentation test, *E. phylloplanum* can grow on glucose but does not ferment it (Suppl. material 10). Thus, fermentation of additional carbon sources was not tested. Growth tests showed that *E. phylloplanum* can grow on all tested carbon sources, although the strains differ slightly in growth rates (Suppl. material 10).

##### Additional specimens examined.

CZECHIA • Litovelské Pomoraví, Střeň, 49.693167°N, 17.139833°E; 225 m a.s.l., isolated from the intestine of caterpillar *Tischeria ekebladella*, May 2018, D. Višňovská (CCF 6536); GenBank sequences: ITS = PV253747, LSU = PX591250, SSU = PX591255; CZECHIA • Litovelské Pomoraví, Střeň, 49.693167°N, 17.139833°E; 225 m a.s.l., isolated from the intestine of caterpillar *Tischeria ekebladella*, May 2018, D. Višňovská (CCF 6537); GenBank sequences: ITS = PX591259, LSU = PX591252, SSU = PX591257.

##### Geography.

Based on cultivation data, it is known from Czechia, Slovakia (this study), and France (*Palmaria palmata*, see Notes). It is widespread across the globe, typically in the temperate climatic zone, with a handful of findings in the tropics and boreal zone (GlobalFungi, Fig. 3D, Suppl. material 8).

##### Notes.

We consistently detected *E. phylloplanum* on the surface of healthy leaves, which rules out a plant-parasitic life strategy. This pattern indicates that the species exhibits a saprophytic lifestyle, consistent with the yeast-like stages of *Ustilaginomycotina* (Begerow et al. 2006), or alternatively behaves also as an opportunistic mycoparasite, as demonstrated for *Pseudozyma* species (Kitamoto et al. 2019; Steins et al. 2023), or as an endophyte. The morphology of the observed conidia and the type of conidiogenesis agree with the yeast stage of *Exobasidium* (Nagao et al. 2006; Piątek et al. 2012; Brewer et al. 2014; Jiang et al. 2024). *Exobasidium phylloplanum* is diagnosed by the secretion of red-brown pigments identified as pyranonaphthoquinone derivatives, gunacins (Stodůlková et al. 2025). Only a small number of *Exobasidium* species are known from culture. They are typically yellowish or non-pigmented, and we are not aware of any that produce a distinct reddish pigment. Four sequences of undetermined fungi deposited in NCBI GenBank, originating from cultures or envDNA, showed 99.1–99.8% similarity to *E. phylloplanum* and are, therefore, most likely this species. They originated from the macroalga *Palmaria palmata* in France (OR582938, unpublished), house dust in Finland (AM902052, Pitkäranta et al. 2008; FR682357, unpublished), alder leaves from streams in Finland (KT160678, Mykrä et al. 2016), and living leaves of *Nothofagus* sp. in New Zealand (MF976765, unpublished). Interestingly, the viability of *E. phylloplanum* propagules isolated from insect guts suggests potential insect-mediated transfer between plants, as reported for *E. maculosum* by Newell et al. (2023). However, the role of insect vectoring in the spread of *Exobasidium* species warrants further experimentation. *Exobasidium* species are described based on features of their sexual state. Therefore, it may represent an asexual stage of an already described *Exobasidium* species for which no molecular data are available.

## Discussion

Fungi represent the largest group of plant pathogens, causing extensive economic losses in agriculture and forestry worldwide (Anand and Rajeshkumar 2022; Gomdola et al. 2022). *Ustilaginomycotina* comprises mainly plant parasites but also encompasses several asexual lineages known exclusively from their saprotrophic, yeast-like phase (Begerow et al. 2014). Some species in these saprotrophic genera were later linked to their parasitic, sexual forms using molecular tools (Begerow et al. 2000; Wang et al. 2015). This demonstrates that certain parasitic smut fungi can undergo independent saprotrophic growth during their life cycle. *Exobasidium phylloplanum* is one such fungus, identified during its saprotrophic phase, with its associated pathogenic phase not yet observed.

*Exobasidium* comprises biotrophic parasites of plants from the order *Ericales*. Despite their significance in tea and blueberry production, key aspects of their life cycle remain poorly understood. For instance, little is known about how these fungi persist during the off-season of their host plants. This is surprising given that this knowledge is crucial for their effective management. Saprotrophic yeast-like growth of *E. maculosum* on its primary host has been proposed (Ingram et al. 2019). However, it remains unclear how important this saprotrophic phase is in other *Exobasidium* species and whether they can grow independently for extended periods and colonize alternative substrates.

In our study, we identified *Exobasidium* as an integral part of the leaf phyllosphere of five common broad-leaved trees in Czechia, none of which belong to the *Ericales*, using both cultivation and envDNA analyses. From the 12 detected phylotypes, only seven could be assigned to known parasitic species. The remaining five phylotypes had low (<95%) similarity to any sequenced *Exobasidium* species. Among these five phylotypes, the most common, Phyl 9, was isolated in pure culture and described as a new species, *Exobasidium phylloplanum*.

We propose that *Exobasidium* species exhibit a range of life history strategies. At one end of this gradient are dominantly parasitic species, with a short saprophytic phase, which are absent or extremely rare in environmental samples. Further along the gradient are parasitic species that appear to possess a longer-lived and more independent saprotrophic phase, potentially allowing growth on alternative substrates (e.g., *E. arescens*). At the opposite extreme are dominantly saprotrophic species occupying a broad spectrum of substrates (e.g., *E. phylloplanum*; see below).

The majority of *Exobasidium* species are rare or absent in the GlobalFungi database, suggesting that their growth is likely restricted to *Ericales* host plants. However, several species, namely *E. phylloplanum*, *E. arescens*, *E. camelliae*, and *E. vaccinii*, are relatively common in environmental samples. *Exobasidium* taxa, including *E. arescens*, *E. bisporum*, *E. canadense*, *E. japonicum*, *E. miyabei*, and *E. otanianum*, have been occasionally listed as components of phylloplane communities of various tree species in genera such as *Betula* (Ivashchenko et al. 2022), *Fagus* (Cordier et al. 2012), *Fraxinus* (Cross et al. 2017), *Mussaenda* (Qian et al. 2018), *Picea* (Nguyen et al. 2016), *Quercus* (Jumpponen and Jones 2010; Menkis et al. 2022), and *Ulmus* (Marčiulynas et al. 2022).

Data on the occurrence of biotrophic pathogens outside the host, based on envDNA analysis or cultivation, have methodological limitations, as it is not possible to distinguish actively growing cells from dormant propagules, which is especially relevant for wind-dispersed fungi such as *Exobasidium* (Shanmuganathan and Arulpragasam 1966). Nevertheless, with careful experimental design and analysis, valuable insights can be derived from envDNA studies. Our sampling was conducted in riparian forests without ericoid plants, and the nearest agglomeration with possible ornamental *Ericales* plantings was approximately 2 km from the sampling site. If our envDNA metabarcoding captured primarily airborne, non-active spores, then the stochastic nature of wind-borne dispersal (Stockmarr et al. 2007) should result in an even distribution of *Exobasidium* phylogenetic lineages across the phyllospheres of different trees. However, our data show that *Exobasidium* communities are significantly non-random and are shaped by host tree identity, with several phylotypes displaying clear host preferences. Consequently, some phylotypes exhibit clear host preferences. This host-driven pattern aligns with established findings on phylloplane microbial communities, where tree species, through their distinct chemical, structural, and microclimatic traits, filter microbial taxa and give rise to characteristic, host-specific assemblages (Šigut et al. 2022; Lajoie and Dariel 2025). Together, these findings indicate that the detected *Exobasidium* does not merely represent passively deposited airborne spores but actively proliferates on the leaf surface.

We hypothesize that some *Exobasidium* species possess an ecologically significant saprotrophic phase in their life cycle, during which they can grow on various alternative host plants. Among the phylotypes identified in our study, the parasitic phase has been recorded in Europe for *E. arescens* and *E. gracile* (Kokeš and Müller 2004; Compagnoni et al. 2024) but not for *E. bisporum*, *E. miyabei*, and *E. maculosum*. *Exobasidium bisporum* and *E. miyabei* are known only from Asia (Ezuka 1991; Nagao et al. 2003), and *E. maculosum* is known only from the USA (Brewer et al. 2014). However, *E. bisporum* and *E. miyabei* were identified as significant species in the phyllosphere communities of *Picea abies* in Lithuania (Menkis et al. 2015) and *Betula pendula* in Moscow, Russia (Ivashchenko et al. 2022), respectively. It cannot be conclusively asserted that these parasites occur in Czechia, as the haplotypes belonging to envDNA phylotypes were more represented in the GlobalFungi database than those of the reference sequences. This suggests they may represent predominantly saprotrophic lineages. Our findings, therefore, highlight intriguing ecological dynamics within this fungal genus and indicate that some *Exobasidium* species possess a saprotrophic phase outside host plants, suggesting a broader ecological role than previously thought. Finally, the saprotrophic life-history strategy on various alternative host plants may be favored by selection as a means of survival when a suitable primary host for the parasitic life-history strategy is lacking, for example, locally after spore dispersal or due to the extinction of a particular host species.

The taxonomic identity of the phylotypes showing less than 95% sequence similarity to any molecularly described *Exobasidium* species remains unclear. Globally, approximately 150 accepted *Exobasidium* species and varieties are listed in the Index Fungorum database (accessed 19 February 2025), yet only 54 of these have publicly available molecular data (NCBI Taxonomy database, 19 February 2025). Thus, many validly described species still lack DNA barcodes. Consequently, we cannot exclude the possibility that our detected phylotypes belong to some of these species. The high sequence similarity of these phylotypes to uncultured fungal sequences from environmental samples in the NCBI database suggests the presence of substantial yet hidden diversity within the genus *Exobasidium*.

Phylotype 9 was particularly abundant in phyllosphere communities. We isolated it into pure culture and describe it here as *E. phylloplanum*, a new species that, like *E. lijiangense* (Jiang et al. 2024), has an unknown pathogenic phase. Based on our data and public data from the GlobalFungi database, *E. phylloplanum* is widespread in nature on various substrates, outside ericoid plants. There are two likely hypotheses about its life cycle. In the first hypothesis, we propose that the parasitic phase is inconspicuous and has been overlooked so far or that it belongs to a described species that has not yet been sequenced. The close relatives of *E. phylloplanum*, *E. woronichinii*, and *E. rhododendri* are parasitic on *Rhododendron*. As co-speciation between *Exobasidium* and its host plants has been proposed (Begerow et al. 2002), *Rhododendron* is the most probable primary host plant for *E. phylloplanum*. However, *Rhododendron* does not naturally occur in Central Europe (Gibbs et al. 2011), except in geographically limited alpine regions. Thus, native *Rhododendron* species do not appear to be plausible hosts for *E. phylloplanum* in Central Europe, where this fungus is relatively common. However, it is possible that its primary host plant is an ornamental *Rhododendron* shrub. Based on corresponding geographic distributions, the other possible host plant could be *Vaccinium vitis-idaea* (Hirabayashi et al. 2024), which largely overlaps with the distribution of *E. phylloplanum*. However, this host does not follow the current proposal of co-speciation between *Exobasidium* and host plants. Here, there is also a discrepancy in the notable absence of *E. phylloplanum* in Scotland and northern Scandinavia, where cranberries and other ericoid plants (including planted *Rhododendron* spp.) are common. It should also be noted that the pathogenic phase may be restricted to certain parts of the species’ geographic range, potentially outside Europe, in regions where *Rhododendron* hosts are more abundant. Further infection experiments are required to assess its parasitic potential.

The second hypothesis is that some *Exobasidium* species, including *E. phylloplanum*, may have secondarily lost their pathogenic stage. In our study, we compared the genome of *E. phylloplanum* with those of four other *Exobasidium* species and two other *Exobasidiales* species, *Meira miltonrushii* (*Brachybasidiaceae*) and *Acaromyces ingoldii* (*Cryptobasidiaceae*). Although *Meira* and *Acaromyces* are currently known only from their haploid, non-parasitic stage, their parasitism cannot be ruled out. Both taxa still retain genes associated with mating and the formation of a sexual, and thus potentially pathogenic, phase (Coelho et al. 2017; Steins et al. 2023). We did not identify any genomic differences that could account for their potentially contrasting life-history strategies. The only notable distinction was that *E. phylloplanum* contains a higher number of type I polyketide synthases (T1PKSs). In line with this, novel secondary metabolites with antiprotozoal activity, the gunacins, were recently described from this species (Stodůlková et al. 2025). A very similar result was obtained from a recent comparative genomic study of saprotrophic *Pseudozyma* and related parasitic species within *Ustilaginales* (Steins et al. 2023). The study shows that genomes of these *Pseudozyma* species bear genes necessary for pathogenesis, which makes the ecology of these species puzzling. It is unclear whether the transition from parasitic to saprotrophic occurred so recently that there has not been enough time for gene loss or whether these species do, in fact, cause mild symptoms that go unnoticed (Steins et al. 2023). So far, it is not known whether genes related to pathogenesis are present or lost in the other asexual saprotrophic lineages. Further research is needed to clarify the implications of these taxa possessing the genetic capacity for pathogenesis, despite no observed evidence linking them to disease thus far.

## Conclusion

Our findings reveal that some *Exobasidium* species can spend part of their life cycle growing on various plants other than their known *Ericales* hosts. They further suggest that some *Exobasidium* species may be predominantly, or theoretically even exclusively, saprotrophic. The former is well documented for *Ustilaginomycotina* (Begerow et al. 2014), but it is undescribed in *Exobasidium* so far. Therefore, as part of our study, we provide the genome of *E. phylloplanum*, which will enable more detailed genome-wide comparative studies in this genus in the future. This will also allow future studies to explore pathogenicity-related genomic traits and, ultimately, virulence factors and specific life-history strategy adaptations. We also encourage further exploration of environmental samples for the presence of *Exobasidium* species to deepen understanding of the ecology of this genus.

## Supplementary Material

XML Treatment for Exobasidium phylloplanum

## Additional information

### Use of AI

The following AI tools were used in the preparation of this manuscript (select all that apply): Description: The language was corrected using an AI assistant, Grammarly Pro.

### Adherence to national and international regulations

All the fungal strains used in this study have been legally obtained, respecting the Convention on Biological Diversity (Rio Convention).

### Author ORCIDs

Tereza Veselská https://orcid.org/0000-0003-4873-6917

Tereza Ježková https://orcid.org/0009-0002-3926-6781

Nombuso P. Ngubane https://orcid.org/0000-0002-0910-2623

Martin Kostovčík https://orcid.org/0000-0001-6982-1470

Denisa Hařovská https://orcid.org/0000-0002-6060-9454

Martin Šigut https://orcid.org/0000-0003-4876-9794

Petr Pyszko https://orcid.org/0000-0002-3743-7201

Ryan Bracewell https://orcid.org/0000-0003-2678-4758

Pavel Drozd https://orcid.org/0000-0002-4602-8856

Dominik Begerow https://orcid.org/0000-0002-8286-1597

Miroslav Kolařík https://orcid.org/0000-0003-4016-0335

### Data availability

All of the data that support the findings of this study are available in the main text or Supplementary Information.

## Supplementary materials

Supplementary material 1 ASV table of fungal taxa retrieved from leaf phylloplan and caterpillar gut mycobiome communities Data type xlsx Explanation note Host plants or caterpillar species, along with their feeding strategies is provided for each sample. https://binary.pensoft.net/file/1562974This dataset is made available under the Open Database License (http://opendatacommons.org/licenses/odbl/1.0/). The Open Database License (ODbL) is a license agreement intended to allow users to freely share, modify, and use this Dataset while maintaining this same freedom for others, provided that the original source and author(s) are credited. Tereza Veselská, Tereza Ježková, Nombuso P. Ngubane, Adéla Wennrich, Martin Kostovčík, Denisa Hařovská, Martin Šigut, Petr Pyszko, Ryan Bracewell, Pavel Drozd, Dominik Begerow, Miroslav Kolařík

Supplementary material 2 ASV table of fungal taxa retrieved from leaf phylloplane and caterpillar gut mycobiome communities after rarefaction Data type xlsx https://binary.pensoft.net/file/1562975This dataset is made available under the Open Database License (http://opendatacommons.org/licenses/odbl/1.0/). The Open Database License (ODbL) is a license agreement intended to allow users to freely share, modify, and use this Dataset while maintaining this same freedom for others, provided that the original source and author(s) are credited. Tereza Veselská, Tereza Ježková, Nombuso P. Ngubane, Adéla Wennrich, Martin Kostovčík, Denisa Hařovská, Martin Šigut, Petr Pyszko, Ryan Bracewell, Pavel Drozd, Dominik Begerow, Miroslav Kolařík

Supplementary material 3 Alignment of ITS sequences Data type fas Explanation note The alignment includes 26 sequences of *Exobasidium* ASVs found in the present study, and 50 sequences of *Exobasidium* strains, and outgroup sequences of *Tilletiopsis pallescens* and *Laurobasidium hachijoense* obtained from NCBI database. Alignment was performed in MAFFT v.7 (Katoh and Standley 2013) using default settings. https://binary.pensoft.net/file/1562976This dataset is made available under the Open Database License (http://opendatacommons.org/licenses/odbl/1.0/). The Open Database License (ODbL) is a license agreement intended to allow users to freely share, modify, and use this Dataset while maintaining this same freedom for others, provided that the original source and author(s) are credited. Tereza Veselská, Tereza Ježková, Nombuso P. Ngubane, Adéla Wennrich, Martin Kostovčík, Denisa Hařovská, Martin Šigut, Petr Pyszko, Ryan Bracewell, Pavel Drozd, Dominik Begerow, Miroslav Kolařík

Supplementary material 4 Alignment of concatenated ITS and LSU sequences for 51 *Exobasidium* strains Data type fas Explanation note *Tilletiopsis pallescens* and *Laurobasidium hachijoense* were used as an outgroup. Alignment was performed in MAFFT v.7 (Katoh and Standley 2013) using default settings. https://binary.pensoft.net/file/1562977This dataset is made available under the Open Database License (http://opendatacommons.org/licenses/odbl/1.0/). The Open Database License (ODbL) is a license agreement intended to allow users to freely share, modify, and use this Dataset while maintaining this same freedom for others, provided that the original source and author(s) are credited. Tereza Veselská, Tereza Ježková, Nombuso P. Ngubane, Adéla Wennrich, Martin Kostovčík, Denisa Hařovská, Martin Šigut, Petr Pyszko, Ryan Bracewell, Pavel Drozd, Dominik Begerow, Miroslav Kolařík

Supplementary material 5 Custom protein database used for genome annotations Data type rar Explanation note List of species strains is provided with their NCBI accession numbers, and related metadata, and custom protein database built from the genomes. https://binary.pensoft.net/file/1562978This dataset is made available under the Open Database License (http://opendatacommons.org/licenses/odbl/1.0/). The Open Database License (ODbL) is a license agreement intended to allow users to freely share, modify, and use this Dataset while maintaining this same freedom for others, provided that the original source and author(s) are credited. Tereza Veselská, Tereza Ježková, Nombuso P. Ngubane, Adéla Wennrich, Martin Kostovčík, Denisa Hařovská, Martin Šigut, Petr Pyszko, Ryan Bracewell, Pavel Drozd, Dominik Begerow, Miroslav Kolařík

Supplementary material 6 Shannon and Simpson indexes of diversity for individual datasets Data type docx Explanation note Mean values with standard deviation are reported. https://binary.pensoft.net/file/1562979This dataset is made available under the Open Database License (http://opendatacommons.org/licenses/odbl/1.0/). The Open Database License (ODbL) is a license agreement intended to allow users to freely share, modify, and use this Dataset while maintaining this same freedom for others, provided that the original source and author(s) are credited. Tereza Veselská, Tereza Ježková, Nombuso P. Ngubane, Adéla Wennrich, Martin Kostovčík, Denisa Hařovská, Martin Šigut, Petr Pyszko, Ryan Bracewell, Pavel Drozd, Dominik Begerow, Miroslav Kolařík

Supplementary material 7 Bonferrroni-corrected p-values obtained from one -way ANOSIM analyses Data type docx https://binary.pensoft.net/file/1562980This dataset is made available under the Open Database License (http://opendatacommons.org/licenses/odbl/1.0/). The Open Database License (ODbL) is a license agreement intended to allow users to freely share, modify, and use this Dataset while maintaining this same freedom for others, provided that the original source and author(s) are credited. Tereza Veselská, Tereza Ježková, Nombuso P. Ngubane, Adéla Wennrich, Martin Kostovčík, Denisa Hařovská, Martin Šigut, Petr Pyszko, Ryan Bracewell, Pavel Drozd, Dominik Begerow, Miroslav Kolařík

Supplementary material 8 Biogeography assessment using GlobalFungi database Data type xlsx Explanation note Search output showing exact matches against the database, including geographic distribution, substrate, biome, and climatic preferences. The number of studies and samples along with total and relative read frequencies is reported for each species. https://binary.pensoft.net/file/1562981This dataset is made available under the Open Database License (http://opendatacommons.org/licenses/odbl/1.0/). The Open Database License (ODbL) is a license agreement intended to allow users to freely share, modify, and use this Dataset while maintaining this same freedom for others, provided that the original source and author(s) are credited. Tereza Veselská, Tereza Ježková, Nombuso P. Ngubane, Adéla Wennrich, Martin Kostovčík, Denisa Hařovská, Martin Šigut, Petr Pyszko, Ryan Bracewell, Pavel Drozd, Dominik Begerow, Miroslav Kolařík

Supplementary material 9 Macromorphology of *Exobasidium phylloplanum* growing on MEA Data type xlsx Explanation note *. phylloplanum* (strains CCF 6536, CCF 6537, CCF 7021) was cultivated for three weeks on MEA, bar = 10 cm. The pigmentation of colonies is visible after one week of cultivation. In the second week, the pigmentation penetrates the growth media. The pigmentation of mature colonies has an orange-brown color in strains CCF 6536 and CCF 7021, while CCF 6537 has pink-violet pigmentation. https://binary.pensoft.net/file/1562982This dataset is made available under the Open Database License (http://opendatacommons.org/licenses/odbl/1.0/). The Open Database License (ODbL) is a license agreement intended to allow users to freely share, modify, and use this Dataset while maintaining this same freedom for others, provided that the original source and author(s) are credited. Tereza Veselská, Tereza Ježková, Nombuso P. Ngubane, Adéla Wennrich, Martin Kostovčík, Denisa Hařovská, Martin Šigut, Petr Pyszko, Ryan Bracewell, Pavel Drozd, Dominik Begerow, Miroslav Kolařík

Supplementary material 10 Fermentation and carbon assimilation tests of *E. phylloplanum* Data type jpg Explanation note All three strains of *E. phylloplanum*, CCF 6536, CCF 6537, CCF 7021, were used. Saccharomyces cerevisiae served as positive control for fermentation tests, while *E. vaccinii* for assimilation tests. https://binary.pensoft.net/file/1562983This dataset is made available under the Open Database License (http://opendatacommons.org/licenses/odbl/1.0/). The Open Database License (ODbL) is a license agreement intended to allow users to freely share, modify, and use this Dataset while maintaining this same freedom for others, provided that the original source and author(s) are credited. Tereza Veselská, Tereza Ježková, Nombuso P. Ngubane, Adéla Wennrich, Martin Kostovčík, Denisa Hařovská, Martin Šigut, Petr Pyszko, Ryan Bracewell, Pavel Drozd, Dominik Begerow, Miroslav Kolařík

Supplementary material 11 Number of predicted proteins within individual functional groups Data type docx https://binary.pensoft.net/file/1562984This dataset is made available under the Open Database License (http://opendatacommons.org/licenses/odbl/1.0/). The Open Database License (ODbL) is a license agreement intended to allow users to freely share, modify, and use this Dataset while maintaining this same freedom for others, provided that the original source and author(s) are credited. Tereza Veselská, Tereza Ježková, Nombuso P. Ngubane, Adéla Wennrich, Martin Kostovčík, Denisa Hařovská, Martin Šigut, Petr Pyszko, Ryan Bracewell, Pavel Drozd, Dominik Begerow, Miroslav Kolařík
