# Supplementary material for: The saprotrophic dimension of Exobasidium (Exobasidiales, Basidiomycota): evidence for greater diversity and ecological flexibility than previously recognized
Source: IMA Fungus. 2026 Mar 16;17:e180524. doi: 10.3897/imafungus.17.180524 (PMC13010171; doi:10.3897/imafungus.17.180524)
Supplement: Supplementary material 6 — Shannon and Simpson indexes of diversity for individual datasets [file imafungus-17-e180524-s006.docx]

Supplementary material 6. Shannon and Simpson indexes of diversity for individual datasets.

| *Exobasidium* community in | *A. glutinosa –*  phyllosphere | *Ca. betulus –*  phyllosphere | *Co. avellana –*  phyllosphere | *Q. petraea –*  phyllosphere | *Q. robur –*  phyllosphere |
| --- | --- | --- | --- | --- | --- |
| Shannon index | 0.37 ± 0.34 | 0.48 ± 0.70 | 0.41 ± 0.33 | 0.50 ± 0.68 | 0.32 ± 0.25 |
| Simpson index | 0.22 ± 0.23 | 0.41 ± 0.27 | 0.18 ± 0.23 | 0.39 ± 0.26 | 0.17 ± 0.21 |
| *Exobasidium* community in | *A. glutinosa –*  caterpillar gut | *C. betulus –*  caterpillar gut | *Co. avellana –*  caterpillar gut | *Q. petraea –*  caterpillar gut | *Q. robur –*  caterpillar gut |
| Shannon index | 0.75 ± 0.53 | 0.34 ± 0.43 | 0.69 ± 0.35 | 0.35 ± 0.32 | 0.84 ± 0.39 |
| Simpson index | 0.41 ± 0.28 | 0.2 ± 0.26 | 0.42 ± 0.17 | 0.21 ± 0.20 | 0.48 ± 0.22 |

Mean values with standard deviation are reported.
