# Supplementary material for: The saprotrophic dimension of Exobasidium (Exobasidiales, Basidiomycota): evidence for greater diversity and ecological flexibility than previously recognized
Source: IMA Fungus. 2026 Mar 16;17:e180524. doi: 10.3897/imafungus.17.180524 (PMC13010171; doi:10.3897/imafungus.17.180524)
Supplement: Supplementary material 7 — Bonferrroni-corrected p-values obtained from one -way ANOSIM analyses [file imafungus-17-e180524-s007.docx]

**Supplementary material 7: Bonferroni-corrected *p*-values obtained from one -way ANOSIM analyses.**

Analyses of *Exobasidium* community in tree phylloshperes. The right upper triangle (yellow) represents the Morista distance measure, while the lower left triangle (green) represents the Jaccard distance measure.

|  | *A. glutinosa* | *Ca. betulus* | *Co. avellana* | *Q. petraea* | *Q. robur* |
| --- | --- | --- | --- | --- | --- |
| *A. glutinosa* | - | 0.001 | 0.001 | 0.001 | 1.000 |
| *Ca. betulus* | 0.001 | - | 0.001 | 0.001 | 0.004 |
| *Co. avellana* | 0.001 | 0.001 | - | 0.001 | 0.001 |
| *Q. petraea* | 0.001 | 0.001 | 0.001 | - | 0.001 |
| *Q. robur* | 1.000 | 0.009 | 0.001 | 0.003 | - |

Analyses of *Exobasidium* community in caterpillar guts. Right upper triangle (yellow) represents Morista distance measure. while lower left tringle (green) represents Jaccard distance measure.

|  | *A. glutinosa* | *Ca. betulus* | *Co. avellana* | *Q. petraea* | *Q. robur* |
| --- | --- | --- | --- | --- | --- |
| *A. glutinosa* | - | 0.130 | 1.00 | 0.840 | 1.000 |
| *Ca. betulus* | 0.259 | - | 0.135 | 0.397 | 0.476 |
| *Co. avellana* | 1.000 | 0.444 | - | 0.154 | 1.000 |
| *Q. petraea* | 0.395 | 0.170 | 0.244 | - | 1.000 |
| *Q. robur* | 1.000 | 0.112 | 1.00 | 1.0000 | - |
