# Supplementary material for: The saprotrophic dimension of Exobasidium (Exobasidiales, Basidiomycota): evidence for greater diversity and ecological flexibility than previously recognized
Source: IMA Fungus. 2026 Mar 16;17:e180524. doi: 10.3897/imafungus.17.180524 (PMC13010171; doi:10.3897/imafungus.17.180524)
Supplement: Supplementary material 11 — Number of predicted proteins within individual functional groups [file imafungus-17-e180524-s011.docx]

Supplementary material 11. Number of predicted proteins within individual functional groups.

|  | *E. cylindrosporum* (YG638) | *E. maculosum* (A7-4) | *E. phylloplanum* (CCF7021) | *E. rhododendri* (CBS101457) | *E. vaccinii* (MPITM) |
| --- | --- | --- | --- | --- | --- |
| CAZymes | 250 | 295 | 268 | 244 | 224 |
| GH | 107 | 103 | 112 | 94 | 83 |
| GT | 80 | 122 | 89 | 96 | 89 |
| PL | 3 | 3 | 2 | 3 | 2 |
| CE | 22 | 25 | 21 | 16 | 16 |
| AA | 35 | 39 | 41 | 30 | 32 |
| CBM | 3 | 3 | 3 | 5 | 2 |
| Secondary metabolite clusters | 17 | 13 | 23 | 13 | 11 |
| NRPS-like | 5 | 5 | 6 | 5 | 4 |
| NRPS | 4 | 2 | 3 | 3 | 1 |
| terpen | 4 | 4 | 4 | 4 | 4 |
| terpen-precursor | 0 | 0 | 2 | 0 | 0 |
| NI-siderophore | 1 | 0 | 1 | 0 | 0 |
| NAPAA | 1 | 0 | 1 | 1 | 0 |
| T1PKS | 2 | 2 | 5 | 0 | 1 |
| indole | 0 | 0 | 1 | 0 | 0 |
| fungal RiPP | 0 | 0 | 0 | 0 | 1 |
| Signal proteins | 845 | 1309 | 708 | 667 | 725 |
| Intracellular | 187 | 456 | 145 | 240 | 184 |
| Membrane associated | 176 | 207 | 189 | 140 | 175 |
| Extracellular | 313 | 417 | 243 | 205 | 246 |
| Effector | 169 | 229 | 131 | 82 | 120 |
| Pathogen host interaction | 2963 | 3699 | 3065 | 2808 | 2935 |
| Increased virulence | 30 | 43 | 31 | 26 | 27 |
| Reduced virulence | 664 | 890 | 692 | 670 | 680 |
| Loss of pathogenicity | 65 | 87 | 66 | 68 | 69 |
| Lethal | 41 | 54 | 46 | 43 | 43 |
| Effector | 50 | 55 | 54 | 43 | 44 |
| Unaffected | 314 | 426 | 326 | 310 | 336 |
| Conflict* | 1829 | 2187 | 1881 | 1674 | 1763 |

CAZymes – carbohydrate-active enzymes, GH – glycoside hydrolases, GT – glycosyl transferases, PL – polysaccharide lyases, CE – carbohydrate esterases, AA – auxiliary activities, CBM – carbohydrate binding modules. * more than one function was found.
